# [4-Bromo-*N*-(pyridin-2-yl­methyl­idene)aniline-κ^2^ *N*,*N*′]bis­(1,1,1,5,5,5-hexa­fluoro­pentane-2,4-dionato-κ^2^ *O*,*O*′)nickel(II)

**DOI:** 10.1107/S1600536811005599

**Published:** 2011-03-09

**Authors:** Phimphaka Harding, David J. Harding, Nitisastr Soponrat, Harry Adams

**Affiliations:** a Molecular Technology Research Unit, Department of Chemistry, Walailak University, Thasala, Nakhon Si Thammarat 80161, Thailand; b Department of Chemistry, Faculty of Science, Taksin University, Songkhla 90000, Thailand; c Department of Chemistry, Faculty of Science, University of Sheffield, Brook Hill, Sheffield S3 7HF, England

## Abstract

The title compound, [Ni(C_5_HF_6_O_2_)_2_(C_12_H_9_BrN_2_)], the Ni^II^ atom exhibits a pseudo-octa­hedral coordination geometry. The structure packs through C—H⋯Br inter­actions, forming a hydrogen-bonded ladder. There are also strong hydrogen-bonding inter­actions between two of the O atoms of the β-diketonate ligands and two H atoms on the pyridine ring of the Schiff base ligand.

## Related literature

For related structures, see: Harding, Harding, Sophonrat & Adams (2010 ▶); Harding, Harding, Tinpun *et al.* (2010 ▶); Aakeröy *et al.* (2004 ▶, 2005 ▶, 2007 ▶). For an introduction to crystal engineering, see: Braga *et al.* (2002 ▶). For details concerning the coordination of additional ligands to β-diketon­ate complexes, see: Chassot & Emmenegger (1996 ▶); Emmenegger *et al.* (2001 ▶). For a description of the Cambridge Structural database, see: Allen *et al.* (2002 ▶).
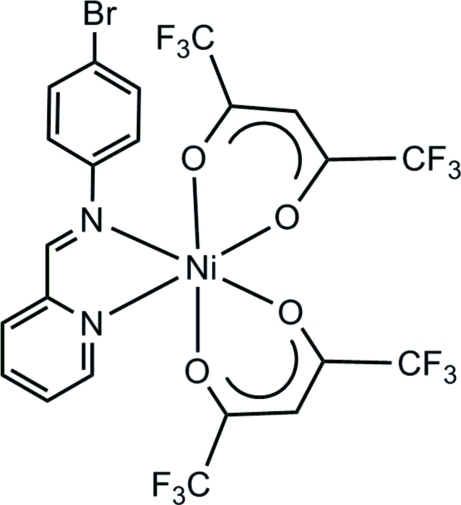


## Experimental

### 

#### Crystal data

[Ni(C_5_HF_6_O_2_)_2_(C_12_H_9_BrN_2_)]
*M* *_r_* = 733.95
Monoclinic, 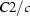

*a* = 31.251 (8) Å
*b* = 10.006 (3) Å
*c* = 17.653 (5) Å
β = 103.952 (5)°
*V* = 5358 (2) Å^3^
*Z* = 8
Mo *K*α radiation
μ = 2.33 mm^−1^
*T* = 150 K
0.22 × 0.12 × 0.11 mm

#### Data collection

Bruker SMART CCD area-detector diffractometer
Absorption correction: multi-scan (*SADABS*; Bruker, 1997 ▶) *T*_min_ = 0.628, *T*_max_ = 0.784
27645 measured reflections
5988 independent reflections
3422 reflections with *I* > 2σ(*I*)
*R*_int_ = 0.145

#### Refinement

*R*[*F* ^2^ > 2σ(*F* ^2^)] = 0.054
*wR*(*F* ^2^) = 0.132
*S* = 0.91
5988 reflections
379 parameters
H-atom parameters constrained
Δρ_max_ = 1.24 e Å^−3^
Δρ_min_ = −1.12 e Å^−3^

### 

Data collection: *SMART* (Bruker, 1997 ▶); cell refinement: *SAINT* (Bruker, 1997 ▶); data reduction: *SAINT*; program(s) used to solve structure: *SHELXS97* (Sheldrick, 2008 ▶); program(s) used to refine structure: *SHELXL97* (Sheldrick, 2008 ▶); molecular graphics: *SHELXTL* (Sheldrick, 2008 ▶); software used to prepare material for publication: *SHELXTL* and *publCIF* (Westrip, 2010 ▶).

## Supplementary Material

Crystal structure: contains datablocks I, global. DOI: 10.1107/S1600536811005599/zb2010sup1.cif

Structure factors: contains datablocks I. DOI: 10.1107/S1600536811005599/zb2010Isup2.hkl

Additional supplementary materials:  crystallographic information; 3D view; checkCIF report

## Figures and Tables

**Table 1:** Selected bond lengths (Å)

Ni1—O3	2.020 (3)
Ni1—O4	2.044 (3)
Ni1—O2	2.045 (3)
Ni1—N2	2.063 (3)
Ni1—O1	2.066 (3)
Ni1—N1	2.113 (4)

**Table 2:** Hydrogen-bond geometry (Å, °)

*D*—H⋯*A*	*D*—H	H⋯*A*	*D*⋯*A*	*D*—H⋯*A*
C6—H6⋯Br1^i^	0.95	3.02	3.870 (3)	151
C2—H2⋯Br1^ii^	0.95	3.02	3.833 (3)	145
C12—H12⋯O1^iii^	0.95	2.53	3.320 (4)	140
C11—H11⋯O4^iii^	0.95	2.61	3.470 (3)	151

Symmetry codes: (i) 
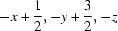
; (ii) 
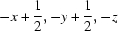
; (iii) 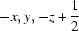.

**Fig. 1.**
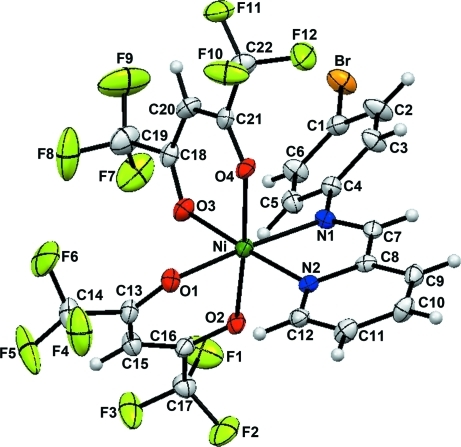
The molecular structure of (1) showing the atom-labelling scheme. Displacement ellipsoids are drawn at the 50% probability level.

**Fig. 2.**
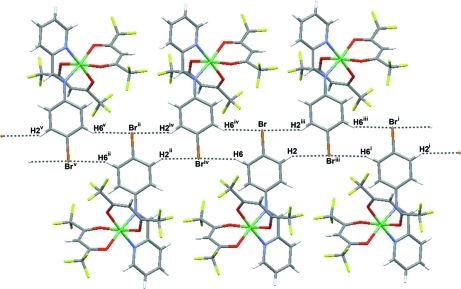
The molecular packing in (1) showing the C—H···Br interactions between neighbouring ppaBr ligands. Only selected atoms are labelled for clairty. [Symmetry codes: (i) x, -1 + y, z; (ii) x, 1 + y, z; (iii) 1/2 - x, 1/2 - y, -z; (iv) 1/2 - x, 3/2 - y, -z; (v) 1/2 - x, 5/2 - y, -z].

**Fig. 3.**
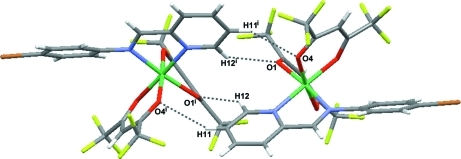
The molecular packing in (1) showing the O···H interactions between the oxygen atoms of the β-diketonate ligands and the two hydrogen of the ppaBr ligand. Only selected atoms are labelled for clairty. [Symmetry codes: (i) -x, y, 1/2 - z].

## supplementary crystallographic information

### Comment

Metal β-diketonates represent an important class of complexes and are much studied owing to their ease of synthesis, ready modification and multiple applications. In the case of divalent metal ions, the [*M*(β-diketonate)_2_] complexes are able to coordinate additional ligands forming either *cis-* or *trans-*octahedral metal complexes (Chassot & Emmenegger, 1996; Emmenegger *et al.*, 2001). Of particular relevence to this paper is the use metal β-diketonates complexes in the preparation of crystal engineered networks (Braga *et al.*, 2002) and while hydrogen bonded *trans*-isomers are well represented few compounds containing *cis*-isomers are described (Aäkeroy *et al.*, 2004, 2005, 2007). In this paper we describe the synthesis and structure of [Ni(hfac)_2_(ppa^Br^)] (hfac = 1,1,1,5,5,5-hexafluoropentane-2,4-dionato; ppa^Br^ = (4-bromo-phenyl)pyridin-2-ylmethylene amine).

[Ni(hfac)_2_(H_2_O)_2_] reacts readily with ppa^Br^ to give [Ni(hfac)_2_(ppa^Br^)] 1 which recrystallizes from CH_2_Cl_2_/*n*-hexane to give yellow crystals in the space group *C*2/c (Figure 1). This contrasts markedly with the analogous cobalt compound which crystallizes in *P*1 (Harding, Harding, Sophonrat and Adams, 2010). The nickel metal centre is *pseudo*-octahedral with a *cis*-arrangement enforced by the chelating ppa^Br^ ligand. The Ni—O and Ni—N bond lengths are typical of values reported for other nickel hfac and diimine complexes reported in the CSD (mean Ni—O distance = 2.01 Å, Ni—N distance = 2.11 Å, Allen, 2002). The β-diketonate ligands exhibit a *bent* coordination mode in which the angles between the planes defined by the Ni and oxygen atoms and the carbon and oxygen atoms of the β-diketonate ligand are 11.0° and 26.8°. In contrast, in *trans*-[*M*(hfac)_2_(py-CH=CH—C_6_F_4_Br)_2_] (*M* = Co, Cu) the β-diketonate ligands exhibit a *planar* coordination mode (Aäkeroy *et al.*, 2007). In addition, the phenyl ring is twisted with to the pyridylimine unit by 30.0° a little greater than the angle observed in [Ni(dbm)_2_(ppa*^X^*)] (*X* = Me 22.9°, Cl 24.0°, Harding, Harding, Tinpun *et al.*, 2010).

The packing in the structure is composed of two sets of interactions. The first set is involves a series of C—H···Br interactions (H6···Br 3.015 (3) Å, H2···Br 3.017 (3) Å) forming a hydrogen bonding ladder (Figure 2). The second interaction involves a strong hydrogen bonding interactions between two of the oxygen atoms of the β-diketonate ligands and two hydrogen atoms on the pyridyl ring of the ppa^Br^ ligand (O1···H12 2.532 (4) Å, O4···H11 2.610 (3) Å) forming a dimer (Figure 3). In contrast, the cobalt analogue has extensive π···π interactions and interactions between the Br atom on the ppa^Br^ ligand and the β-diketonate ligand (Harding, Harding, Sophonrat and Adams, 2010).

### Experimental

To a green solution of [Ni(hfac)_2_(H_2_O)_2_] (0.128 g, 0.25 mmol) in CH_2_Cl_2_ (10 ml) was added a solution of ppa^Br^ (0.065 g, 0.25 mmol) in CH_2_Cl_2_ (3 ml). The orange solution was stirred overnight then concentrated *in vacuo. n-*Hexane (10 ml) was added to precipitate a yellow brown solid which was washed with *n*-hexane (2 *x* 5 ml) and dried *in vacuo* yielding a deep yellow solid (0.096 g, 52%). Found: C 36.2, H 1.7, N 3.9. Calc. for C_22_H_11_BrF_12_N_2_NiO_4_: C 36.0, H 1.5, N 3.8%. *m*/*z* (ESI) 527 [*M*-hfac^-^]^+^. n_max_(KBr)/cm^-1^ 1651 (n_C=O_). l_max_(CH_2_Cl_2_)/nm (log e/*M*^-1^cm^-1^) 319 (4.46), 342 (4.34). Mpt. 156 °C.

### Refinement

Hydrogen atoms were placed geometrically and refined with a riding model and with *U*_iso_ constrained to be 1.2 (aromatic CH) times *U*_eq_ of the carrier atom.

### Crystal data

**Table:**

[Ni(C_5_HF_6_O_2_)_2_(C_12_H_9_BrN_2_)]	*F*(000) = 2880
*M**_r_* = 733.95	*D*_x_ = 1.820 Mg m^−^^3^
Monoclinic, *C*2/*c*	Melting point: 429 K
Hall symbol: -C2yc	Mo *K*α radiation, λ = 0.71073 Å
*a* = 31.251 (8) Å	Cell parameters from 3767 reflections
*b* = 10.006 (3) Å	θ = 2.4–24.9°
*c* = 17.653 (5) Å	µ = 2.33 mm^−^^1^
β = 103.952 (5)°	*T* = 150 K
*V* = 5358 (2) Å^3^	Block, brown
*Z* = 8	0.22 × 0.12 × 0.11 mm

### Data collection

**Table:**

Bruker SMART CCD area-detector diffractometer	5988 independent reflections
Radiation source: fine-focus sealed tube	3422 reflections with *I* > 2σ(*I*)
graphite	*R*_int_ = 0.145
Detector resolution: 100 pixels mm^-1^	θ_max_ = 27.5°, θ_min_ = 1.3°
φ and ω scans	*h* = −39→40
Absorption correction: multi-scan (*SADABS*; Bruker, 1997)	*k* = −12→12
*T*_min_ = 0.628, *T*_max_ = 0.784	*l* = −22→22
27645 measured reflections	

### Refinement

**Table:**

Refinement on *F*^2^	Primary atom site location: structure-invariant direct methods
Least-squares matrix: full	Secondary atom site location: difference Fourier map
*R*[*F*^2^ > 2σ(*F*^2^)] = 0.054	Hydrogen site location: inferred from neighbouring sites
*wR*(*F*^2^) = 0.132	H-atom parameters constrained
*S* = 0.91	*w* = 1/[σ^2^(*F*_o_^2^) + (0.0546*P*)^2^] where *P* = (*F*_o_^2^ + 2*F*_c_^2^)/3
5988 reflections	(Δ/σ)_max_ < 0.001
379 parameters	Δρ_max_ = 1.24 e Å^−^^3^
0 restraints	Δρ_min_ = −1.12 e Å^−^^3^

### Special details

**Table:**

Geometry. All e.s.d.'s (except the e.s.d. in the dihedral angle between two l.s. planes) are estimated using the full covariance matrix. The cell e.s.d.'s are taken into account individually in the estimation of e.s.d.'s in distances, angles and torsion angles; correlations between e.s.d.'s in cell parameters are only used when they are defined by crystal symmetry. An approximate (isotropic) treatment of cell e.s.d.'s is used for estimating e.s.d.'s involving l.s. planes.
Refinement. Refinement of *F*^2^ against ALL reflections. The weighted *R*-factor *wR* and goodness of fit *S* are based on *F*^2^, conventional *R*-factors *R* are based on *F*, with *F* set to zero for negative *F*^2^. The threshold expression of *F*^2^ > σ(*F*^2^) is used only for calculating *R*-factors(gt) *etc*. and is not relevant to the choice of reflections for refinement. *R*-factors based on *F*^2^ are statistically about twice as large as those based on *F*, and *R*- factors based on ALL data will be even larger.

### Fractional atomic coordinates and isotropic or equivalent isotropic displacement parameters (Å^2^)

**Table:**

	*x*	*y*	*z*	*U*_iso_*/*U*_eq_	
Ni1	0.089498 (18)	0.60018 (5)	0.17271 (3)	0.01947 (16)	
Br1	0.279804 (16)	0.50379 (5)	−0.01356 (3)	0.03493 (16)	
N1	0.10544 (11)	0.4874 (3)	0.0821 (2)	0.0180 (8)	
N2	0.03045 (12)	0.5037 (3)	0.1283 (2)	0.0193 (8)	
O1	0.06741 (10)	0.7013 (3)	0.25796 (16)	0.0227 (7)	
O2	0.07571 (10)	0.7678 (3)	0.10485 (16)	0.0206 (7)	
O3	0.14882 (9)	0.6873 (3)	0.21444 (17)	0.0253 (7)	
O4	0.10989 (9)	0.4413 (3)	0.24516 (16)	0.0215 (7)	
C1	0.22491 (15)	0.4979 (4)	0.0144 (3)	0.0268 (11)	
C2	0.20348 (15)	0.3757 (5)	0.0139 (3)	0.0318 (12)	
H2	0.2157	0.2965	−0.0018	0.038*	
C3	0.16424 (15)	0.3716 (4)	0.0365 (3)	0.0269 (11)	
H3	0.1497	0.2885	0.0375	0.032*	
C4	0.14579 (14)	0.4876 (4)	0.0578 (3)	0.0197 (10)	
C5	0.16748 (15)	0.6093 (4)	0.0566 (3)	0.0257 (11)	
H5	0.1549	0.6892	0.0707	0.031*	
C6	0.20726 (14)	0.6144 (4)	0.0351 (3)	0.0236 (10)	
H6	0.2221	0.6971	0.0347	0.028*	
C7	0.07464 (14)	0.4036 (4)	0.0519 (3)	0.0222 (10)	
H7	0.0788	0.3410	0.0139	0.027*	
C8	0.03340 (13)	0.4058 (4)	0.0765 (2)	0.0168 (9)	
C9	−0.00087 (15)	0.3178 (4)	0.0481 (3)	0.0255 (11)	
H9	0.0019	0.2505	0.0116	0.031*	
C10	−0.03943 (15)	0.3295 (4)	0.0736 (3)	0.0261 (11)	
H10	−0.0632	0.2691	0.0560	0.031*	
C11	−0.04250 (15)	0.4301 (5)	0.1249 (3)	0.0265 (11)	
H11	−0.0687	0.4407	0.1426	0.032*	
C12	−0.00710 (14)	0.5167 (4)	0.1508 (3)	0.0223 (10)	
H12	−0.0098	0.5870	0.1855	0.027*	
C13	0.07108 (15)	0.8261 (4)	0.2653 (2)	0.0236 (10)	
C14	0.06750 (18)	0.8781 (5)	0.3451 (3)	0.0326 (12)	
C15	0.07746 (15)	0.9195 (4)	0.2105 (3)	0.0249 (11)	
H15	0.0810	1.0110	0.2250	0.030*	
C16	0.07882 (15)	0.8822 (4)	0.1348 (3)	0.0241 (11)	
C17	0.08415 (17)	0.9929 (4)	0.0773 (3)	0.0278 (11)	
C18	0.18236 (15)	0.6331 (4)	0.2566 (3)	0.0227 (10)	
C19	0.22211 (17)	0.7250 (6)	0.2750 (3)	0.0410 (14)	
C20	0.18587 (15)	0.5046 (4)	0.2881 (3)	0.0281 (11)	
H20	0.2140	0.4740	0.3162	0.034*	
C21	0.14966 (15)	0.4192 (4)	0.2797 (3)	0.0232 (10)	
C22	0.15671 (15)	0.2806 (5)	0.3172 (3)	0.0286 (11)	
F1	0.11909 (12)	0.9686 (3)	0.0484 (2)	0.0590 (10)	
F2	0.04965 (11)	0.9967 (3)	0.01655 (16)	0.0474 (9)	
F3	0.08890 (12)	1.1150 (3)	0.10758 (16)	0.0516 (9)	
F4	0.03084 (12)	0.8339 (3)	0.3627 (2)	0.0651 (10)	
F5	0.06729 (15)	1.0080 (3)	0.35147 (18)	0.0703 (12)	
F6	0.09967 (13)	0.8311 (4)	0.40128 (18)	0.0775 (13)	
F7	0.22453 (10)	0.8019 (3)	0.2159 (2)	0.0584 (10)	
F8	0.21997 (13)	0.8058 (4)	0.3339 (2)	0.0789 (13)	
F9	0.26008 (10)	0.6604 (4)	0.2971 (3)	0.0827 (14)	
F10	0.13059 (10)	0.2608 (3)	0.3655 (2)	0.0540 (9)	
F11	0.19792 (9)	0.2582 (3)	0.35786 (17)	0.0395 (8)	
F12	0.14753 (11)	0.1857 (3)	0.26264 (18)	0.0522 (9)	

### Atomic displacement parameters (Å^2^)

**Table:**

	*U*^11^	*U*^22^	*U*^33^	*U*^12^	*U*^13^	*U*^23^
Ni1	0.0209 (3)	0.0193 (3)	0.0168 (3)	−0.0020 (2)	0.0019 (2)	−0.0002 (2)
Br1	0.0284 (3)	0.0261 (3)	0.0550 (4)	−0.0007 (2)	0.0192 (3)	−0.0007 (2)
N1	0.0176 (19)	0.0186 (19)	0.020 (2)	0.0011 (15)	0.0087 (16)	0.0019 (15)
N2	0.019 (2)	0.0198 (18)	0.0171 (19)	0.0022 (15)	0.0002 (16)	0.0014 (15)
O1	0.0265 (17)	0.0234 (17)	0.0181 (16)	−0.0041 (13)	0.0051 (14)	−0.0022 (13)
O2	0.0269 (17)	0.0229 (16)	0.0123 (15)	−0.0012 (13)	0.0052 (14)	0.0002 (12)
O3	0.0202 (17)	0.0289 (17)	0.0248 (17)	−0.0049 (14)	0.0014 (15)	−0.0015 (14)
O4	0.0239 (17)	0.0250 (16)	0.0134 (15)	−0.0003 (13)	0.0001 (14)	0.0030 (13)
C1	0.023 (3)	0.026 (2)	0.028 (3)	0.003 (2)	0.000 (2)	0.001 (2)
C2	0.021 (3)	0.025 (3)	0.055 (4)	0.002 (2)	0.019 (2)	0.000 (2)
C3	0.024 (3)	0.018 (2)	0.041 (3)	−0.0019 (18)	0.013 (2)	0.000 (2)
C4	0.020 (2)	0.020 (2)	0.019 (2)	−0.0013 (18)	0.005 (2)	0.0001 (18)
C5	0.031 (3)	0.023 (2)	0.024 (2)	0.000 (2)	0.008 (2)	−0.002 (2)
C6	0.019 (2)	0.025 (2)	0.032 (3)	−0.0018 (19)	0.016 (2)	0.002 (2)
C7	0.028 (3)	0.016 (2)	0.019 (2)	0.0015 (19)	0.000 (2)	0.0013 (18)
C8	0.016 (2)	0.021 (2)	0.014 (2)	0.0027 (18)	0.0062 (18)	0.0056 (18)
C9	0.030 (3)	0.019 (2)	0.023 (2)	−0.0042 (19)	−0.001 (2)	−0.0012 (19)
C10	0.020 (3)	0.035 (3)	0.023 (3)	−0.008 (2)	0.005 (2)	−0.001 (2)
C11	0.023 (3)	0.038 (3)	0.020 (2)	−0.001 (2)	0.007 (2)	0.002 (2)
C12	0.022 (3)	0.027 (2)	0.017 (2)	0.0056 (19)	0.003 (2)	0.0011 (18)
C13	0.029 (3)	0.024 (2)	0.017 (2)	−0.005 (2)	0.003 (2)	−0.0033 (19)
C14	0.049 (3)	0.027 (3)	0.024 (3)	−0.012 (2)	0.012 (3)	−0.005 (2)
C15	0.031 (3)	0.020 (2)	0.025 (3)	−0.0018 (19)	0.009 (2)	−0.0032 (19)
C16	0.025 (3)	0.019 (2)	0.023 (2)	−0.0035 (18)	−0.003 (2)	0.0025 (19)
C17	0.034 (3)	0.024 (2)	0.026 (3)	−0.001 (2)	0.009 (2)	0.001 (2)
C18	0.025 (3)	0.029 (3)	0.015 (2)	−0.005 (2)	0.007 (2)	−0.0012 (19)
C19	0.030 (3)	0.044 (3)	0.046 (4)	−0.011 (3)	0.003 (3)	0.005 (3)
C20	0.020 (2)	0.034 (3)	0.027 (3)	0.001 (2)	0.000 (2)	0.002 (2)
C21	0.026 (3)	0.026 (2)	0.016 (2)	0.002 (2)	0.002 (2)	−0.0045 (19)
C22	0.023 (3)	0.033 (3)	0.030 (3)	0.001 (2)	0.008 (2)	0.003 (2)
F1	0.064 (2)	0.051 (2)	0.075 (3)	0.0070 (17)	0.042 (2)	0.0280 (18)
F2	0.059 (2)	0.0475 (19)	0.0271 (16)	−0.0053 (15)	−0.0060 (16)	0.0152 (14)
F3	0.100 (3)	0.0219 (15)	0.0304 (17)	−0.0123 (16)	0.0116 (18)	0.0018 (13)
F4	0.077 (3)	0.076 (2)	0.058 (2)	−0.030 (2)	0.045 (2)	−0.0352 (19)
F5	0.158 (4)	0.0290 (18)	0.0383 (19)	−0.0167 (19)	0.052 (2)	−0.0129 (14)
F6	0.086 (3)	0.113 (3)	0.0216 (17)	0.031 (2)	−0.0094 (19)	−0.0215 (19)
F7	0.050 (2)	0.066 (2)	0.056 (2)	−0.0321 (17)	0.0068 (18)	0.0199 (18)
F8	0.081 (3)	0.090 (3)	0.069 (3)	−0.051 (2)	0.022 (2)	−0.045 (2)
F9	0.0247 (18)	0.076 (3)	0.136 (4)	−0.0113 (18)	−0.004 (2)	0.041 (3)
F10	0.046 (2)	0.054 (2)	0.072 (2)	0.0198 (15)	0.0331 (19)	0.0345 (17)
F11	0.0330 (16)	0.0378 (16)	0.0419 (18)	0.0070 (13)	−0.0021 (15)	0.0108 (13)
F12	0.068 (2)	0.0274 (16)	0.050 (2)	0.0015 (15)	−0.0074 (18)	−0.0033 (15)

### Geometric parameters (Å, °)

**Table:**

Ni1—O3	2.020 (3)	C9—H9	0.9500
Ni1—O4	2.044 (3)	C10—C11	1.372 (6)
Ni1—O2	2.045 (3)	C10—H10	0.9500
Ni1—N2	2.063 (3)	C11—C12	1.393 (6)
Ni1—O1	2.066 (3)	C11—H11	0.9500
Ni1—N1	2.113 (4)	C12—H12	0.9500
Br1—C1	1.897 (5)	C13—C15	1.394 (6)
N1—C7	1.291 (5)	C13—C14	1.531 (7)
N1—C4	1.426 (6)	C14—F5	1.304 (5)
N2—C12	1.332 (6)	C14—F6	1.317 (6)
N2—C8	1.358 (5)	C14—F4	1.332 (6)
O1—C13	1.258 (5)	C15—C16	1.399 (6)
O2—C16	1.255 (5)	C15—H15	0.9500
O3—C18	1.253 (5)	C16—C17	1.538 (6)
O4—C21	1.265 (5)	C17—F2	1.326 (5)
C1—C6	1.376 (6)	C17—F3	1.327 (5)
C1—C2	1.393 (6)	C17—F1	1.334 (6)
C2—C3	1.379 (6)	C18—C20	1.394 (6)
C2—H2	0.9500	C18—C19	1.517 (7)
C3—C4	1.388 (6)	C19—F7	1.313 (6)
C3—H3	0.9500	C19—F9	1.325 (6)
C4—C5	1.396 (6)	C19—F8	1.331 (7)
C5—C6	1.386 (6)	C20—C21	1.397 (6)
C5—H5	0.9500	C20—H20	0.9500
C6—H6	0.9500	C21—C22	1.529 (6)
C7—C8	1.456 (6)	C22—F10	1.330 (6)
C7—H7	0.9500	C22—F12	1.333 (5)
C8—C9	1.384 (6)	C22—F11	1.333 (5)
C9—C10	1.390 (7)		
			
O3—Ni1—O4	89.27 (12)	C11—C10—C9	118.7 (4)
O3—Ni1—O2	84.55 (12)	C11—C10—H10	120.7
O4—Ni1—O2	173.82 (12)	C9—C10—H10	120.7
O3—Ni1—N2	177.27 (13)	C10—C11—C12	119.8 (5)
O4—Ni1—N2	89.36 (12)	C10—C11—H11	120.1
O2—Ni1—N2	96.80 (12)	C12—C11—H11	120.1
O3—Ni1—O1	87.79 (12)	N2—C12—C11	121.9 (4)
O4—Ni1—O1	91.93 (12)	N2—C12—H12	119.1
O2—Ni1—O1	87.99 (12)	C11—C12—H12	119.1
N2—Ni1—O1	94.62 (13)	O1—C13—C15	128.3 (4)
O3—Ni1—N1	98.24 (13)	O1—C13—C14	114.1 (4)
O4—Ni1—N1	87.91 (12)	C15—C13—C14	117.7 (4)
O2—Ni1—N1	92.82 (12)	F5—C14—F6	108.1 (4)
N2—Ni1—N1	79.35 (14)	F5—C14—F4	106.8 (5)
O1—Ni1—N1	173.97 (13)	F6—C14—F4	104.4 (4)
C7—N1—C4	120.1 (4)	F5—C14—C13	114.9 (4)
C7—N1—Ni1	111.8 (3)	F6—C14—C13	111.0 (4)
C4—N1—Ni1	127.9 (3)	F4—C14—C13	111.0 (4)
C12—N2—C8	118.6 (4)	C13—C15—C16	121.8 (4)
C12—N2—Ni1	127.8 (3)	C13—C15—H15	119.1
C8—N2—Ni1	113.2 (3)	C16—C15—H15	119.1
C13—O1—Ni1	121.6 (3)	O2—C16—C15	129.0 (4)
C16—O2—Ni1	121.2 (3)	O2—C16—C17	112.9 (4)
C18—O3—Ni1	126.3 (3)	C15—C16—C17	118.1 (4)
C21—O4—Ni1	124.0 (3)	F2—C17—F3	106.8 (4)
C6—C1—C2	121.5 (5)	F2—C17—F1	106.0 (4)
C6—C1—Br1	119.2 (3)	F3—C17—F1	107.4 (4)
C2—C1—Br1	119.2 (3)	F2—C17—C16	111.0 (4)
C3—C2—C1	118.9 (4)	F3—C17—C16	114.7 (4)
C3—C2—H2	120.5	F1—C17—C16	110.5 (4)
C1—C2—H2	120.5	O3—C18—C20	127.6 (4)
C2—C3—C4	120.7 (4)	O3—C18—C19	112.9 (4)
C2—C3—H3	119.7	C20—C18—C19	119.5 (4)
C4—C3—H3	119.7	F7—C19—F9	107.5 (5)
C3—C4—C5	119.4 (4)	F7—C19—F8	106.8 (5)
C3—C4—N1	122.4 (4)	F9—C19—F8	106.1 (5)
C5—C4—N1	118.2 (4)	F7—C19—C18	112.7 (4)
C6—C5—C4	120.5 (4)	F9—C19—C18	113.3 (4)
C6—C5—H5	119.8	F8—C19—C18	110.0 (5)
C4—C5—H5	119.8	C18—C20—C21	122.6 (4)
C1—C6—C5	119.0 (4)	C18—C20—H20	118.7
C1—C6—H6	120.5	C21—C20—H20	118.7
C5—C6—H6	120.5	O4—C21—C20	128.4 (4)
N1—C7—C8	119.5 (4)	O4—C21—C22	112.9 (4)
N1—C7—H7	120.3	C20—C21—C22	118.7 (4)
C8—C7—H7	120.3	F10—C22—F12	107.4 (4)
N2—C8—C9	122.1 (4)	F10—C22—F11	107.0 (4)
N2—C8—C7	114.9 (4)	F12—C22—F11	106.3 (4)
C9—C8—C7	123.0 (4)	F10—C22—C21	111.5 (4)
C8—C9—C10	119.0 (4)	F12—C22—C21	110.5 (4)
C8—C9—H9	120.5	F11—C22—C21	113.9 (4)
C10—C9—H9	120.5		

### Hydrogen-bond geometry (Å, °)

**Table:**

*D*—H···*A*	*D*—H	H···*A*	*D*···*A*	*D*—H···*A*
C6—H6···Br1^i^	0.95	3.02	3.870 (3)	151
C2—H2···Br1^ii^	0.95	3.02	3.833 (3)	145
C12—H12···O1^iii^	0.95	2.53	3.320 (4)	140
C11—H11···O4^iii^	0.95	2.61	3.470 (3)	151

Symmetry codes: (i) −*x*+1/2, −*y*+3/2, −*z*; (ii) −*x*+1/2, −*y*+1/2, −*z*; (iii) −*x*, *y*, −*z*+1/2.
